# The Prognostic Role of Procalcitonin in Critically Ill Patients Admitted in a Medical Stepdown Unit: A Retrospective Cohort Study

**DOI:** 10.1038/s41598-020-61457-6

**Published:** 2020-03-11

**Authors:** Vincenzo Zaccone, Lorenzo Falsetti, Cinzia Nitti, Tamira Gentili, Annalisa Marchetti, Maria Novella Piersantelli, Mattia Sampaolesi, Francesca Riccomi, Alessia Raponi, Aldo Salvi

**Affiliations:** Internal and Emergency Medicine Department, Azienda Ospedaliero-Universitaria “Ospedali Riuniti”, Ancona, Italy

**Keywords:** Disease-free survival, Predictive markers, Prognostic markers

## Abstract

Procalcitonin (PCT) is a a marker of bacterial infection. Its prognostic role in the critically-ill patient, however, is still object of debate. Aim of this study was to evaluate the capacity of admission PCT (aPCT) in assessing the prognosis of the critically-ill patient regardless the presence of bacterial infection. A single-cohort, single-center retrospective study was performed evaluating critically-ill patients admitted to a stepdown care unit. Age, sex, Simplified Acute Physiology Score II (SAPS-II), shock, troponin-I, aPCT, serum creatinine, cultures and clinical endpoints (in-hospital mortality or Intensive Care Unit (ICU) transfer) were collected. Time free from adverse event (TF-AE) was defined as the time between hospitalization and occurrence of one of the clinical endpoints, and calculated with Kaplan-Meier curves. We engineered a new predictive model (POCS) adopting aPCT, age and shock.We enrolled 1063 subjects: 450 reached the composite outcome of death or ICU transfer. aPCT was significantly higher in this group, where it predicted TF-AE both in septic and non-septic patients. aPCT and POCS showed a good prognostic performance in the whole sample, both in septic and non-septic patients. aPCT showed a good prognostic accuracy, adding informations on the rapidity of clinical deterioration. POCS model reached a performance similar to SAPS-II.

## Introduction

Procalcitonin (PCT) is a pro-hormone of human calcitonin synthesized by the parafollicular C cells of the thyroid and involved in calcium homeostasis. PCT is produced in large quantities by different cell types in patients affected by bacterial infections^1^. Several studies have shown low- to moderately-elevated PCT levels also among critically-ill patients without evidence of infection (trauma, major surgery, multi-organ failure and myocardial infarction)^2,3^.

Clinically, PCT represents a quantitative biomarker which is currently used to help predicting the probability of bacterial infections^4^ and guide the duration of antibiotic therapy^5^. PCT demonstrated not only a diagnostic, but also an elevated prognostic value in septic patients. A recent meta-analysis concluded that increased PCT concentrations and absence of PCT clearance were strongly associated with all-cause mortality in septic patients^6^.

Clinical and pathophysiological data suggest a role of PCT in prognostic evaluation of critically-ill patients regardless the presence of a bacterial infection^7^, but large and conclusive data are still lacking.

Main objective of the present study was to assess the prognostic value of PCT, evaluated as a single assay at the admission (aPCT) within the first 12 hours, in critically-ill patients regardless of the presence of bacterial infection. We also engineered a new predictive model, named “Procalcitonin and Other Clinical Score” (POCS), adopting both clinical and laboratoristic variables (aPCT, age and shock), to improve the prediction of outcomes and compared its performances with other validated prognostic markers, as Troponin I (TnI)^8^ and the Simplified Acute Physiology Score II (SAPS-II)^9^.

## Materials and Methods

This single-cohort observational retrospective study was performed in a 25-beds Internal Medicine with stepdown beds unit (SDU) of the University-Hospital “Azienda Ospedaliero Universitaria Ospedali Riuniti” of Ancona (Italy). Since January 01^st^ 2002 our department adopted an electronic medical record system (EMR) for inpatients’ management: this system contained all the patient’s data, including history, medical visit, systolic and diastolic blood pressure at admission and at discharge, heart rate at admission and at discharge, respiratory rate at admission and at discharge, all the performed electrocardiograms, cardiac and blood pressure monitor results, blood exams at admission and during the SDU stay, discharge diagnosis, comorbidities and outcomes.

All subjects or their caregivers gave their informed consent to the analysis of their data at the moment of the admission and were treated according the Helsinki declaration and following the guidelines current at the moment of the study. The Ethics Committee of Marche Region (CERM) reviewed and approved the study protocol (CERM protocol reference: 2019/7).

### Study setting and population

Our SDU, according to the common definition^10^, admits patients from the emergency department (ED) if affected by severe medical pathologies requiring an intermediate level of care, such as continuous electrocardiographic monitoring, inotropic or vasopressor support, non-invasive ventilation and renal replacement therapy, but still not necessitate of invasive ventilation and ICU care.

The commonly treated conditions in this setting and considered for the present work are: acute heart failure, acute coronary syndromes, pulmonary embolism, acute respiratory failure, acute kidney injury, pancreatitis, sepsis, septic shock, and complicated infections (such as diverticulitis, pneumonia, pyelonephritis, cholecystitis and pleural empyema). These conditions were present alone or in combination in this set of patients.

### Inclusion and exclusion criteria

All the consecutive patients admitted in a 36-month period (from 1^st^ January 2008 to 31^th^ December 2010) were screened from the EMR. A total of 6562 patients was evaluated.

Currently, there is no univocal definition of critical illness: for this reason, in order to outline the critically ill subjects, we selected all the patients affected by a life-threatening condition with signs of physiological deterioration^11^, defined as the presence of at least one abnormal vital sign and one end-organ dysfunction at the admission.

Thus, patients were enrolled if they met at least one of the following clinical inclusion criteria: (1) Glasgow Coma Scale <12^12^; (2) PaO2/FiO2 < 300, respiratory rate <8/min or>20/min, accessory muscle use^13^; (3) mean arterial pressure <70 mmHg, urine output <0,5 mL/kg/hr, use of inotrope or vasopressors^14,15^.

Exclusion criteria were represented by: (1) age <18 years; (2) absence of critical illness, defined as absence of organ dysfunction; (3) data incompleteness at the moment of the study, particularly (4) absence of serum PCT determination within the first 12 hours of admission.

Patients treated with cardiopulmonary resuscitation before the admission, whose PCT values could be significantly raised, are not admitted to our stepdown unit. PCT values could be significantly reduced after dialysis: all measurements were performed at the admission before any dialytic treatment.

### Definitions

We retrospectively collected the following variables: age, sex, presence of shock, admission TnI, aPCT, leucocyte count, aPCT, serum creatinine and biological cultures, if present.

aPCT was defined as the first procalcitonin determination within the first 12 hours from admission to the SDU. Similarly, TnI was defined as the first determination within the first 12 hours from SDU admission. According current literature, TnI was treated as a generic marker of critical illness^8^ and tested in all the consecutive patients independently of admission diagnosis.

SAPS-II score was calculated for each subject following its original definition^9^ with the admission values.

Presence of shock was collected as a dichotomous variable: shock was outlined in all the patients admitted with a systolic blood pressure <90 mmHg, mean arterial pressure <70 mmHg and clinical signs of tissue hypoperfusion, defined as cutaneous (hypothermia, mottling), renal (urine output <0.5 mL/kg/hr) or neurological (mental state, obtundation, disorientation and confusion)^16^.

We labelled as “infective” all the patients who had at least one clinical or instrumental criterion of active infection, defined as at least one positive culture (blood, sputum, urine, pleural or peritoneal effusion) and/or a radiogical evidence of infection; a definite diagnosis of infection was put by the attending physician according to current guidelines for each infective condition.

Each enrolled patient underwent to a retrospective chart review (EMR, radiological and biological data) by the group of researchers and unclear cases were retrospectively discussed by the senior experts of the department and assigned to the “infective” or “non infective” group.

Serum PCT levels were measured with the method of enzyme-linked fluorescent immunoassay (VIDAS B.R.A.H.M.S. PCT). The upper and lower detection limits were 200 ng/mL and 0.05 ng/mL, respectively.

Serum TnI levels were measured with an high-sensitivity method (Siemens Dimension Vista cardiac TnI assay). The upper and lower detection limits were 200 ng/mL and 0.015 ng/mL, respectively (range encompassing the 99th percentile: 0.00-0.045 ng/mL).

### Clinical endpoints and statistical models

The main clinical endpoint, or adverse event, was defined as the composite of in-hospital mortality o Intensive Care Unit (ICU) transfer. We also evaluated the in-hospital length of stay and the free time from adverse event (TF-AE), defined as the time occurred between hospitalization and the occurrence of the main clinical endpoint, calculated by Kaplan-Meier’s statistics.

We adopted clinical and laboratoristic parameters at the admission in a logistic regression model to engineer a score able to predict in-hospital adverse events. We used the composite endpoint as the main outcome and age, aPCT and presence of shock as main predictors. Variables were treated in binary form with the following cut-offs: 65 years for age, 0.5 ng/mL for aPCT, according to the currently suggested cutoffs^7^ and presence or absence of shock^16^. We named this composite model “Procalcitonin and Others Clinical Score” (POCS).

### Statistical analysis

All the variables were collected in an electronic database. We synthesized each variable with mean, standard deviation and 95% confidence interval if normally distributed, or by median, interquantile range (IQR) or percentile confidence interval in case of non-normal data distribution; continuous variables were compared, in normally distributed variables, with t-test or, in non-normally distributed variables, with Mann-Whitney U test; comparison among more than two populations of continuous data have been performed with analysis of variance; binary and categorical data were compared with the chi-squared test.

Temporal events were described with Kaplan-Meier curves, and the comparison between curves was performed with the log-rank test. Sensitivity and specificity were calculated with the standard method, as their confidence intervals. ROC curve analyses were performed according to the standard procedure, and the comparison between curves has been done with the Z-score test.

Multivariate analysis was performed with a linear logistic regression for continuous and discrete data. The model has been engineered with the assumption of logit(y) = xB (where B was taken from logistic analysis coefficients) and event probability has been calculated with the assumption of prob(y) = 1/(1 + exp-xB).

A difference was deemed as statistically significant if p resulted <0.05 in a 2-tailed test. The statistical analysis was performed with the NCSS 2009 package for Windows systems.

## Results

**Figure 1:**
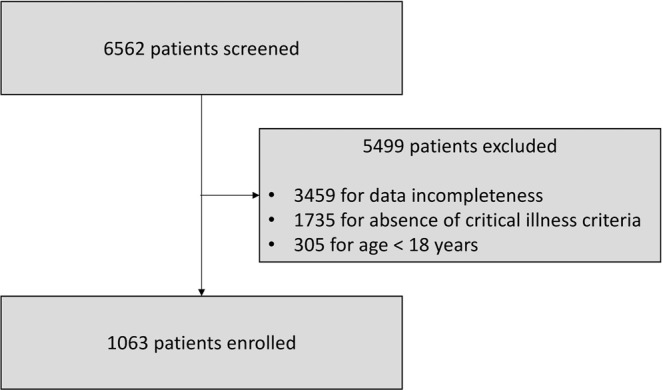
Study flow showing the number of patients included and excluded from the study according to the inclusion and the exclusion criteria.

From an initial screened sample of 6562 patients admitted to the medical SDU of the University-Hospital “Azienda Ospedaliero-Universitaria Ospedali Riuniti” of Ancona (Italy), we excluded 5499 subjects for absence of inclusion criteria or presence of at least one exclusion criteria. The study flow is described in Fig. 1.

We obtained a final sample 1063 critically-ill patients. Baseline characteristics of the sample are summarised in Table 1. At the end of enrolment, we observed that 67.55% of the sample had a definite diagnosis of infection according to validated diagnostic criteria, while the remaining subjects had a “non-infective” critical illness. Diagnoses at the discharge are synthesized in Table 2.

**Table 1:** Baseline characteristics of the study population.

Variable	Value
Sample size (patients)	1063
Age (mean ± SD)	72.4 ± 14.5
Sex (men %)	52%
Days of admission [median, IQR]	8 [5–14]
Death or ICU transfer (%), “worse prognosis” group	450 (42.3%)
Discharge or ordinary department transfer (%), “good prognosis” group	613 (57.7%)
Infective critical illness	718 (67.5%)
Non-infective critical illness	345 (32.5%)
SAPS-II (mean ± SD)	33.1 ± 12.1
Troponin I (mean ± SD), ng/mL	1.238 ± 1.027
Procalcitonin (median, IQR), ng/mL	0.34 [0.06–3.65]
Serum creatinine (mean ± SD), mg/dL	0.86 ± 0.64
Biological cultures (positive %)	35.3% (375)

**Table 2:** Diagnoses at SDU discharge.

Diagnosis	N	%
**NON-INFECTIVE DIAGNOSIS**		
Pulmonary Embolism	28	2.63
Acute Myocardial Infarction	25	2.35
Acute Heart Failure	73	6.86
Pancreatitis	5	0.47
Other non-infective diagnoses	214	20.13
Total	**345**	**32.45**
**INFECTIVE DIAGNOSIS**		
Septic Shock	142	13.35
Diverticolitis	3	0.28
Pneumonia	380	35.74
Pyelonephritis	4	0.37
Colecistitis	14	1.32
Pleural empyema	5	0.47
Other infective diagnoses	170	15.99
Total	**718**	**67.55**

### Clinical end-points

The all-cause in-hospital mortality was 16.8% (n:179); this observation is in concordance with the mean SAPS-II value in the observed population (33.1 ± 12.1), which corresponds to a predicted mortality of 18.1%. The proportion of patients transferred to ICU for clinical deterioration was 25.5% (n:271). We considered the occurrence of ICU transfer as a therapeutic failure and aggregated this event to in-hospital death.

The group of patients who underwent to the composite clinical endpoint (death or ICU transfer) was defined as a “worse prognosis” (WP) group (n:450,42.3%). The remaining subjects, discharged or transferred to non-intensive care departments, were defined as a “good prognosis” (GP) group (n:613,57.7%).

### Prognostic value of aPCT

We observed significantly different aPCT levels according different clinical outcomes: mean aPCT levels were significantly (p < 0.0001) higher in the WP (aPCT:2.39; 95% CI:1.13–3.55) than in the GP group (aPCT:0.23; 95% CI:0.18–0.29).

**Figure 2:**
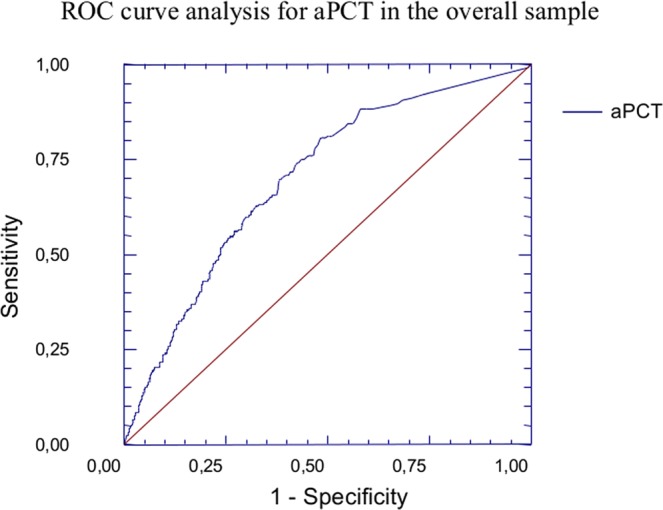
ROC Curve analysis for aPCT in the overall sample. ROC curve analysis showed a good predictive value of aPCT for the composite endpoint (death or transfer in ICU) in the overall sample (AUC:0.690; 95%CI:0.642–0.732; p < 0.05).

**Figure 3:**
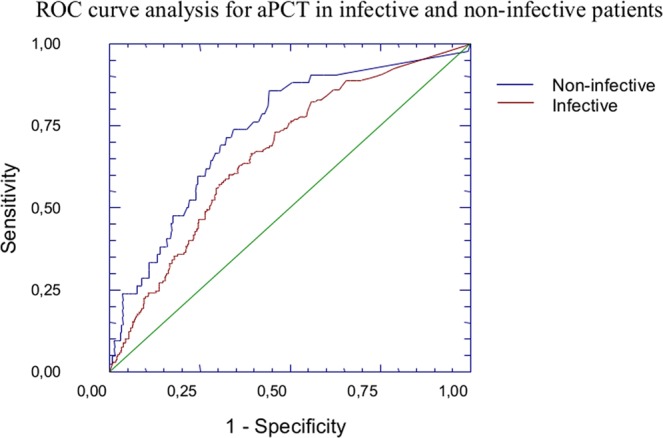
ROC Curve analysis for aPCT in the subgroups of “infective” and “non-infective” patients. ROC curve analysis showed a good predictive value of aPCT for the composite endpoint (death or transfer in ICU) both in the subpopulation of “infective” (AUC:0.660; 95%CI:0.61–0.70; p < 0.05) and “non-infective” patients (AUC:0.732; 95%CI:0.64–0.80; p < 0.05). When comparing the above-mentioned ROC curves, we observed a significantly better performance of the ROC curve in “non-infective” patients (p < 0.05).

ROC curve analysis showed a good predictive value of aPCT for the composite endpoint in the overall sample (AUC:0.690; 95%CI:0.642–0.732; p < 0.05) (Fig. 2), with a fair prognostic capacity both in the subpopulation of “infective” (AUC:0.660; 95%CI:0.61–0.70; p < 0.05) and “non-infective” patients (AUC:0.732; 95%CI:0.64–0.80; p < 0.05), as shown in Fig. 3. When comparing the above-mentioned ROC curves, we observed a significantly better performance of the ROC curve in “non-infective” patients (p < 0.05).

**Figure 4:**
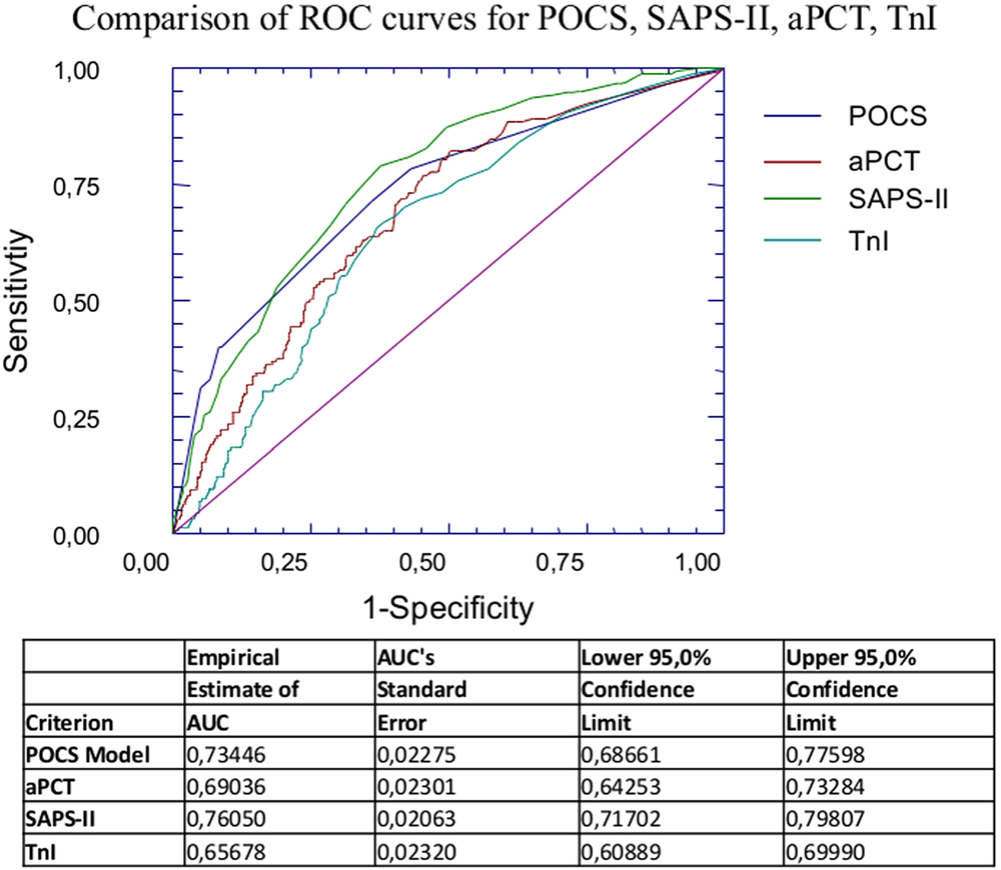
ROC Curve analysis for POCS, SAPS-II, PCT and TnI in the overall sample. In the overall sample, the prognostic performance of aPCT was similar to TnI (AUC_aPCT_ vs AUC_TnI_ p = 0.271) but inferior to SAPS-II (AUC_SAPS-II_ vs AUC_aPCT_ p = 0.0244). We also compared the prognostic performance of POCS model with other indicators as SAPS-II, aPCT or TnI. We did not find any statistically significant difference between AUC_POCS_ and AUC_SAPS-II_ (p = 0.317), while AUC_aPCT_ and AUC_TnI_ were significantly lower than AUC_SAPS-II_ (AUC_aPCT_ vs AUC_SAPS-II_ p = 0.024; AUC_TnI_ vs AUC_SAPS-II_ p = 0.0009).

In the overall sample, the prognostic performance of aPCT was similar to TnI (AUC:0.657; 95%CI:0.609–0.699; p < 0.05) but inferior to SAPS-II (AUC:0.760; 95%CI:0.717–0.798; p < 0.05), as shown in Fig. 4 (AUC_aPCT_ vs AUC_TnI_ p = 0.271; AUC_SAPS-II_ vs AUC_aPCT_ p = 0.0244).

### Composite model of in-hospital mortality

We adopted both clinical and laboratoristic parameters at the admission to engineer a model able to predict in-hospital adverse events. We named this composite model “Procalcitonin and Others Clinical Score” (POCS). In POCS we considered the following parameters: (1) aPCT, taking a cut-off > 0.5 ng/mL as suggested by literature^7^; (2) age, taking a cutoff of 65 years, as derived from literature data^17^; (3) shock criteria, as previously defined by literature^16^. The POCS equation is synthesized in Table 3. Hosmer and Lemeshow test showed no evidence of poor fit of the model (p = 0.241).

**Table 3:** POCS equation.

**Prob(Y)** = 1/[1 + Exp(−XB)]
**Where B** = −1.84638531591793–0.947881848652313*(aPCT) + 0.64398536658017*(age + 1.80028562923787*(shock)

Legend: aPCT = 1 (aPCT < 0.5); PCT = 0 (aPCT > 0.5); age = 0 (<65 years); age = 1 (>65 years); shock = 0 (absence of shock criteria); shock = 1 (presence of shock criteria).

Prognostic performance of this model was good in the overall sample (AUC:0.730; 95%CI:0.680–0.770; p < 0.05), but also in the group of “infective” patients (AUC:0.752; 95%CI:0.705–0.799; p < 0.05) and in the group of “non-infective” subjects (AUC:0.670; 95%CI:0.570–0.769; p < 0.05). The difference between AUC of the “infective” group and the AUC of the “non-infective” group resulted non significant (p = 0.130).

We also compared the prognostic performance of POCS model with other indicators, as SAPS-II, aPCT or TnI comparing their ROC curves (Fig. 3). We did not find any statistically significant difference between AUC_POCS_ and AUC_SAPS-II_ (p = 0.317), while AUC_aPCT_ and AUC_TnI_ were significantly lower than AUC_SAPS-II_ (AUC_aPCT_ vs AUC_SAPS-II_ p = 0.024; AUC_TnI_ vs AUC_SAPS-II_ p = 0.0009).

### aPCT and free time from adverse events

**Figure 5:**
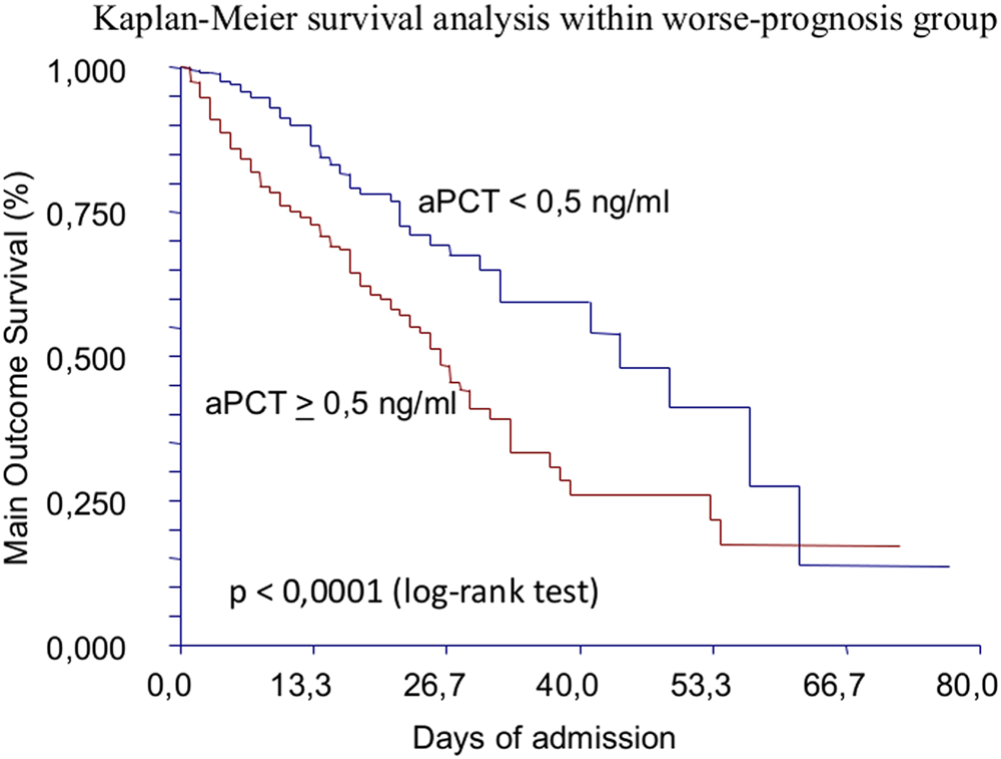
Kaplan-Meier survival analysis within WP group, according to aPCT values and adopting 0.5 ng/mL as cutoff. This curves confirmed that TF-AE can be significantly modified by aPCT levels at a cutoff of 0.50 ng/mL (p < 0.0001).

We considered the WP group and divided it in two subpopulations according their aPCT levels, adopting a cutoff of 0.50 ng/mL. We compared the two populations according the TF-AE endpoint. The subpopulation with aPCT <0.50 ng/mL (n:478) had a median TF-AE of 44 days (95%CI:32–57), while the subgroup of aPCT ≥0.50 ng/mL (n:585) had a median TF-AE of 26 days (95%CI:23–29). Kaplan-Meier curves (Fig. 5) confirmed that TF-AE can be significantly modified by aPCT levels at a cutoff of 0.50 ng/mL (p < 0.0001). Increasing aPCT cutoff to a value of 2.00 ng/mL did not substantially modify the above-mentioned results. For aPCT <2.00 ng/mL (n:744), median TF-AE was 41 days (95%CI:31–54), while for aPCT ≥2.00 ng/mL (n:319) median TF-AE was 25 days (95%CI:20–28).

A definite diagnosis of bacterial infection was present in 61% of the subgroup with aPCT <2.00 ng/mL (n: 455); the number of patients with diagnosis of bacterial infection increased to 82% in the group of subjects with aPCT ≥2.00 ng/mL (n: 263).

Last, we divided the WP group in two subpopulations: “infective WP” (iWP) and “non-infective WP” (niWP). In the iWP group, aPCT significantly predicted TF-AE at a cutoff of 2.00 ng/mL (aPCT < 2.00 ng/mL:TF-AE:44; 95%CI:30–49; aPCT ≥2.00 ng/mL: TF-AE:26; 95%CI:23–29). Similarly, in the niWP population, aPCT was able to modify TF-AE (aPCT < 2.00 ng/mL: TF-AE:not reached; aPCT ≥2 ng/mL:TF-AE:25; 95%CI:16–72).

## Discussion

In this single-cohort, retrospective study we observed that a single determination of aPCT was associated to in-hospital prognosis in a population of critically-ill patients admitted to an Internal Medicine SDU from the ED.

The magnitude of this association is similar to other biomarkers, as admission TnI, but lower than SAPS-II. The elaboration of a composite clinical and laboratoristic model, based on a reducted set of variables, allowed us to improve the prognostic performance of aPCT. Age and shock are recognised prognostic factors for sepsis^18^ that could improve the prediction of infective patients at higher risk of in-hospital adverse events. With this model, aPCT performance was superior to absolute aPCT and admission TnI and similar to SAPS-II.

Our score, however, needs less items than SAPS-II and, if validated in larger cohorts, could be easier to use, especially in patients in critical conditions. aPCT levels were also able to predict the time free from adverse events (death or ICU transfer) in the group of subjects with a worse prognosis.

Several studies have already underlined the ability of serum PCT, both as an absolute value and in terms of non-clearance, to septic patients prognosis^6^. However, PCT levels are increased not only during bacterial infections^1^, but also during other conditions, commonly present in critically-ill patients, such as burns, trauma, necrosis, organ failure and surgery^2,3^.

Thus, we can hypothesise that PCT could have a role in the prognostic evaluation of the patients affected by sepsis or septic shock but also, generally, in all the critically-ill patients, independently of the presence of infection. This has already been postulated and confirmed in other studies: a PCT increase in 24 hours in the critically-ill patient has been associated with an increase of in-hospital mortality at 90 days^7^.

With the present study, we underline the importance of a single PCT determination at the admission in a medical SDU in the prediction of in-hospital prognosis of the critically-ill patient: aPCT levels were significantly higher in patients with worse prognosis, represented by in-hospital death or ICU transfer, than the ones observed in patients with a more favourable outcome. ROC curves shown a good accuracy in the defining the in-hospital prognosis in the whole population and independently of the presence of an infection. Thus, we can postulate that the prognostic ability of aPCT could represent the increase of the systemic inflammation during a critical illness that could finally evolve into a multiorgan failure.

Actually, several composite clinical and instrumental prognostic indices have been validated for the critically-ill patient, as SAPS-II^9^, APACHE II^19^ and MEWS^20^. Some biomarkers, as TnI, have also been associated to a worse prognosis in the critically-ill patient^8^.

Our data underline that an early aPCT determination could represent, alone or in association with clinical features, an easy, economic and fast approach to predict prognosis in this setting, allowing an earlier risk stratification and suggesting a more aggressive diagnostic and therapeutic strategy in the patients at risk.

However, according to our data, an extensive screening of critically-ill subjects with aPCT should be considered only for an accurate risk stratification. aPCT can also be useful to improve diagnosis in several medical conditions, but its results must be carefully interpreted in the setting of patient’s history, physical examination, radiologic and microbiologic tests in order to reduce unnecessary treatment^21^.

The strengths of this work are represented by the large number of subjects and the well-defined population under analysis: to date, this is the largest study evaluating the role of PCT in critically-ill patients. However, this study has several limitations: the retrospective nature of the analysis limits the generalizability of the results. Moreover, due to its single-center design, its results should be validated in prospective, multicenter studies.

## Conclusions

aPCT could represent a potential tool to stratify the risk of adverse events in the critically-ill patient admitted in medical SDU. Our clinical-laboratoristic model, if validated in larger samples, could be easy and useful to stratify earlier patients’ risk of adverse events. These data, in a critical, time-dependent medicine, represent a further implementation of previous studies that correlated the mortality of the patient to a serial evaluation of PCT^7^.
